# Dr. G. V. Black’s Conclusions Reviewed

**Published:** 1897-12

**Authors:** Charles S. Tomes

**Affiliations:** London, England


### DR. G. V. BLACK'S CONCLUSIONS REVIEWED.[1]

BY CHARLES S. TOMES, F.R.S., LONDON, ENGLAND.

The experiments of Dr. Galippe, published in 1884, upon the specific gravities of different teeth, and those published in 1896 by Dr. Black, who appears to have overlooked those of the earlier experimenter, but who has extended his experiments to other

[1] Read before The New York Institute of Stomatology, October 5, 1897.

physical and chemical characters, are both, so far as they go, to be accepted as reliable in the absence of data to the contrary. It is useless to refuse to accept experimental facts, which are probably in the main correct, because they will not square with general impressions.

In the interest of scientific truth I have been compelled (*Journal British Dental Association*, 1895, *Transactions of the Odontological Society*, 1896, and fourth edition "Dental Surgery," 1897) to adversely criticise some of Dr. Black's work, but I wish at the outset to say that I value it very highly in its broad results, although I have a good deal to say in the way of criticism, and I by no means accept all the conclusions which he has drawn from his experiments.

Roughly speaking, I accept his integral figures; his second place of decimals I reject entirely, as being beyond the possible range of experimental accuracy, even had the methods employed been entirely satisfactory, which they were not; for similar reasons I distrust, though in a less degree, his first place of decimals. Hence all the inferences drawn from comparatively small figures I hold to be based upon data which have not been established.

It is impossible to enter at length here into my reasons for distrust, but, lest I seem to be speaking without warrant, I will briefly say why I hold the methods not to have been all that they might have been.

In the first place, Dr. Black weighed "wet" dentine. Now, "wetness" is a manifestly uncertain quantity, and chemists, while recording the amount of water that they can dry out by the prolonged employment of a temperature of 212° F., invariably base their calculated record of analyses on dry material.

In the next place, the dentine was incinerated in little blocks; now, the complete incineration of organic matter in bodies such as bone or dentine is not so easy that the complete combustion of the carbon is readily effected even when the fragments are so small that air has free access to a large part of their surfaces; in a block it is obviously even less exposed to oxygen than when in chips or turnings. Then the blocks were touched, and moved after partial incineration, another source of error in minute weighings; they should have been in platinum crucibles from the first to the last if the utmost attainable accuracy was sought. Hence there were sources of error in all the incineration experiments which, while they would not vitiate the rough results, still prevent us from building hypotheses upon small differences.

Another source of error, also vitiating the accuracy of close weighings, was that no account was taken of the presence of carbonates in the dentine. When you incinerate salts among which carbonates are present you drive off some, but very rarely quite all, of the carbonic acid, thereby diminishing to an uncertain extent the weight of your residue, and therefore it is the rule for chemists to restore the lost carbonic acid before weighing an ash; this was apparently not done. Hence, surprised at Dr. Black's result that there was no difference between the proportion of lime salts present in good and in bad teeth, and seeing that the record of his experiments revealed sources of error, I undertook some check experiments myself, using every care and precaution known to me, with the result of not upsetting but confirming his principal contention, that the difference was not to be sought in the amount of the lime salts present.

For although my results differed a little from his, and in the direction of a slight deficiency in the salts in a set of poor teeth, yet, even if this was not an accident of the particular set, the difference was too small to have much effect. In the dentine of an elephant's tusk there is only fifty-seven per cent. of salts, while in the dentine of its molar there is seventy per cent., and yet the physical difference between the two is relatively slight.

But the inference that the difference as regards liability to caries lies outside the composition of the tooth is very far from being justified; it may be so, but a very much more searching investigation is called for before it is in the least established.

To begin with, to take the salts alone, they may be the same in total quantity and yet be very different in their proportions; a poor tooth may contain much more carbonate and less phosphate than a good one, or the reverse, and yet incineration experiments might not bring out any difference.

Again, we know very little for certain about the phosphate itself; it is not certain what phosphate it is, of the many which exist; and it is not certain if Hoppe Seyler's view be correct, that the hardening salt of bone and dentine and enamel is a double salt, a combination of one equivalent of carbonate with three equivalents of tribasic phosphate, analogous to the mineral apatite.

If Hoppe Seyler's bone salt is a reality, then a little proportional difference in the carbonate or phosphate present would result in the presence of one or other salt in the free condition, and, perhaps, therefore more easily attackable by a solvent.

Again, there is no wet method of preparing the tribasic phosphate anhydrous; it always contains one, two, or more equivalents of water in combination, which cannot be dried out short of red heat; it is possible that different hydrates may exist in different teeth without a difference in the incinerated ash showing it.

As I have elsewhere pointed out (*Transactions of the Odontological Society*, 1896), the analyses of dentine as usually set out are entirely misleading, owing to their being based solely upon incinerations, and so setting down as organic matter all the combined water as well as the real organic matter. Thus, instead of setting down the composition of ivory (dried at 212° F.),—

Lime salts, 57.5;
Organic matter, 42.5 per cent., we ought to write,—

Lime salts, 57.5;
Organic matter, 34;
Water in chemical combination, 8.5 per cent.

The quantities of free and combined water may be a source of difference of quality. It has not escaped Dr. Black that there may also be a chemical difference in the organic matter of the dentine; but there may be a chemical difference in the enamel also, although, according to Mr. Leon Williams, the explanation is not to be sought in the physical peculiarities of the enamel.

To sum up, while Dr. Black's researches have added a material set of facts to our knowledge, the inferences drawn from them are in excess of what they prove, and we cannot by any means safely say that the differences between good and bad teeth have no relation to their calcification; it may be so or it may not, so far as our safe data go.

All that we can say, and so far I fully go with him, is that between good and bad teeth there is not that difference in the total amount of salts that might have been expected, and that the difference in the total of salts, if there be one, is inadequate to produce well-marked effects in its physical characters.

For my own part, I still cherish the conviction that I can recognize by my eye a tooth of good quality and one of bad quality, and though I am compelled to abandon the vague idea which I once had that the bad tooth was deficient in its total salts,—Dr. Black has demonstrated that that notion is untenable,—I am not prepared to give up the idea that there is a physical or chemical difference until the subject has been thrashed out with far greater completeness than is the case at present.

Formerly we knew nothing, but imagined a great deal; now Dr. Galippe and Dr. Black have given us some scraps of positive knowledge to go upon; but it is only repeating our old error of forming hypotheses upon no data to form hypotheses which are far in excess of our data. And though it is not possible in brief compass to enter fully into abstruse matters such as these, yet I have perhaps said enough to justify my contention that, for all we as yet really know, there is plenty of room for physical and chemical differences between teeth of various qualities. Dr. Black has pricked one bubble, and for that I for one am very grateful to him, but inasmuch as his conclusions are somewhat at variance with clinical experience, I would utter a note of warning against the supposition that we so far know more than a very little as to the constitution of dentine and that we are in any position to make wide and general conclusions about it.

I have discussed one portion only of Dr. Black's papers and the chemical aspect of the question, but there is a great deal else of value in his research, and the profession is much indebted to him for a solid contribution to knowledge. If I have seemed to criticise unfavorably, that must be set down to the necessity of brevity and of dwelling rather upon points of disagreement than on those upon which we are in accord.

---

